# Extracellular Vesicles Shed By *Trypanosoma cruzi* Potentiate Infection and Elicit Lipid Body Formation and PGE_2_ Production in Murine Macrophages

**DOI:** 10.3389/fimmu.2018.00896

**Published:** 2018-04-27

**Authors:** Maria Isabel Lovo-Martins, Aparecida Donizette Malvezi, Nágela Ghabdan Zanluqui, Bruno Fernando Cruz Lucchetti, Vera Lúcia Hideko Tatakihara, Patricia Alves Mörking, Admilton Gonçalves de Oliveira, Samuel Goldenberg, Pryscilla Fanini Wowk, Phileno Pinge-Filho

**Affiliations:** ^1^Instituto Carlos Chagas, Fiocruz - Paraná, Curitiba, Brazil; ^2^Laboratório de Imunopatologia Experimental, Departamento de Ciências Patológicas, Centro de Ciências Biológicas, Universidade Estadual de Londrina, Londrina, Brazil; ^3^Laboratório de Microscopia Eletrônica e Microanálises, Central de Laboratórios de Pesquisa Multiusuários, Universidade Estadual de Londrina, Londrina, Brazil; ^4^Laboratório de Virologia Molecular, Instituto Carlos Chagas, Fiocruz - Paraná, Curitiba, Brazil

**Keywords:** extracellular vesicles, lipid bodies, macrophages, prostaglandin E_2_, *Trypanosoma cruzi*

## Abstract

During the onset of *Trypanosoma cruzi* infection, an effective immune response is necessary to control parasite replication and ensure host survival. Macrophages have a central role in innate immunity, acting as an important trypanocidal cell and triggering the adaptive immune response through antigen presentation and cytokine production. However, *T. cruzi* displays immune evasion mechanisms that allow infection and replication in macrophages, favoring its chronic persistence. One potential mechanism is the release of *T. cruzi* strain Y extracellular vesicle (EV Y), which participate in intracellular communication by carrying functional molecules that signal host cells and can modulate the immune response. The present work aimed to evaluate immune modulation by EV Y in C57BL/6 mice, a prototype resistant to infection by *T. cruzi* strain Y, and the effects of direct EV Y stimulation of macrophages *in vitro*. EV Y inoculation in mice prior to *T. cruzi* infection resulted in increased parasitemia, elevated cardiac parasitism, decreased plasma nitric oxide (NO), reduced NO production by spleen cells, and modulation of cytokine production, with a reduction in TNF-α in plasma and decreased production of TNF-α and IL-6 by spleen cells from infected animals. *In vitro* assays using bone marrow-derived macrophages showed that stimulation with EV Y prior to infection by *T. cruzi* increased the parasite internalization rate and release of infective trypomastigotes by these cells. In this same scenario, EV Y induced lipid body formation and prostaglandin E_2_ (PGE_2_) production by macrophages even in the absence of *T. cruzi*. In infected macrophages, EV Y decreased production of PGE_2_ and cytokines TNF-α and IL-6 24 h after infection. These results suggest that EV Y modulates the host response in favor of the parasite and indicates a role for lipid bodies and PGE_2_ in immune modulation exerted by EVs.

## Introduction

The protozoan *Trypanosoma cruzi*, the etiologic agent of Chagas disease, was discovered by Carlos Chagas more than 100 years ago when its life cycle, vector, and pathogenesis in humans were described (1). However, the elucidation of the mechanisms involved in the parasite–host interaction and modulation of the immune response remain incompletely understood. According to the World Health Organization (2017), seven to eight million people are infected with *T. cruzi* worldwide, and over than 10,000 deaths are recorded annually, mostly in Latin America, an endemic area for Chagas disease (2). Recently, new cases have been reported in North America, Asia, and Europe, mainly due to the migration of infected individuals from endemic areas and through non-vectorial transmissions, such as blood transfusion, organ transplants, and congenital transmissions (3).

In vector-borne transmissions, metacyclic trypomastigotes are released in the triatomine excreta when insects feed on mammalian blood. These metacyclic trypomastigotes invade host cells where they transform into replicative amastigotes. After intense replication, amastigotes differentiate into trypomastigotes, an event that culminates with the disruption of the infected cell membrane and release of a substantial number of blood trypomastigote forms that spread the pathogen to other body sites, such as heart tissues (4, 5). During early infection, the innate immune system initiates a host defense that plays an important role in controlling parasite replication and spread in host tissue, with the involvement of cytokines IFN-γ (6), TNF-α (7), and IL-12 (8) and immune cells such as macrophages, natural killers, and dendritic cells (9, 10). Later in the infection, adaptive immunity develops, potentiating innate immunity. A strong and persistent polarized T helper 1 response is established against intracellular parasites (11), and an anti-*T. cruzi* CD8 immune response is focused on parasitic immunodominant epitopes. These T-cell responses are important for controlling parasitemia, tissue parasitism, and polyclonal B-cell activation with antibody production (12). However, due to *T. cruzi* immune evasion mechanisms, this intense immune response fails to clear parasitic infection, leading to its persistence and development of the chronic phase of Chagas disease (13).

An important immune evasion mechanism in infectious diseases is the cell–cell modulatory action promoted by the release of heterogeneous extracellular vesicles (EVs). The following main classes of EVs are categorized based on their intracellular origin: the exosomes (50–150 nm diameter) formed inside multivesicular bodies and released upon fusion of these endosomal compartments with the plasma membrane and plasma membrane-derived EVs (50–1000 nm diameter) that are formed by direct budding and constriction from the plasma membrane. These vesicles can transport proteins from the membrane of the original cell, as well as intracellular proteins and nucleic acids, including small RNAs, which can modulate the target cell (14). Hence, EVs have the potential to be used for immunotherapy, immunization, and diagnosis. In fact, especally in *T. cruzi*, the following different mechanisms for vesicle release have been described: larger vesicles that bud from the plasma membrane and smaller vesicles that bud within the flagellar pocket are released through exocytosis of multivesicular bodies (15, 16).

Recently, the role of EVs that are secreted by different parasites during infection and disease progression have been the focus of several investigations. In some cases, EVs displayed a protective role (17–22). However, in other cases, injection of EVs resulted in downregulation of the immune response followed by an increase in parasitemia (23). Furthermore, when EVs from *Trypanosoma brucei* were engulfed by mammalian erythrocytes, cell clearance led to anemia (24).

Although it has been known since 1991 that shedding *T. cruzi* EVs contain several molecules that interfere with the immune response (25), it was only in 2009 that Trocoli-Torrecilhas et al. showed that inoculation of Balb/c mice, a classical Chagas disease susceptibility model, with EVs from *T. cruzi* strain Y 7 days before infection led to early mortality and severe heart damage (26). *T. cruzi* EVs carry proteins related to metabolism, signaling, survival, and parasite virulence (15, 27). Moreover, small RNA and proteins involved in the small RNA pathway were detected inside EVs (28). Compared with the parasite intercellular compartment, this small RNA has a distinctive profile (29) and varies between parasite stages used to obtain EVs (30). Indeed, *T. cruzi* EVs promote gene expression changes in host cells, mainly modifying host cell cytoskeleton, extracellular matrix, and immune responses pathways, which are essential events in the *T. cruzi*-host cell interplay (31). Moreover, interaction between *T. cruzi* EVs and lineage cells *in vitro* suggests that the release of EVs from *T. cruzi*-infected host cells is important for the modulation of host responses against the parasite (32, 33). These phenomena are strain specific, meaning that EVs shed by host cells infected by one strain do not have effects on the host response to another strain (34).

In this work, we aimed to investigate the role of EVs from *T. cruzi* strain Y in the innate immune compartment, peritoneal macrophages, and spleen cells from C57BL/6 mice during *T. cruzi* acute infection through the quantification of nitric oxide (NO), cytokines, and macrophage immunophenotyping. In addition, we developed *in vitro* experiments using macrophages to assess the influence of EVs released by *T. cruzi* in the interaction between *T. cruzi* and host cells. Understanding the mechanisms by which EVs from the parasite act on host cells could eventually lead to the design of new strategies to control Chagas disease.

## Materials and Methods

### Animals

C57BL/6 mice (male, aged 6–8 weeks) were obtained from the Instituto Carlos Chagas, Curitiba/Fiocruz-PR, Brazil, and maintained under standard conditions in the animal house of the Departamento de Ciências Patológicas, Centro de Ciências Biológicas, Universidade Estadual de Londrina. A commercial rodent diet (Nuvilab-CR1, Quimtia-Nuvital, Colombo, Brazil) and sterilized water were available *ad libitum*.

This study was carried out in accordance with the recommendations of the Guide for the Care and Use of Laboratory Animals of the Brazilian National Council of Animal Experimentation. The protocol was approved by the Committee on the Ethics of Animal Experiments at Londrina State University (CEEA, process number 11/2015- 7045.2015.06). The use of Swiss mice for the maintenance of the parasite strain was also approved for the Committee on the Ethics of Animal Experiments at Londrina State University (CEEA, process number 28.841.2016.41).

### Parasite and Cells

*Trypanosoma cruzi* strain Y, belonging to the *T. cruzi* I (TcI) lineages (35), was maintained by weekly intraperitoneal inoculations of blood trypomastigote forms into Swiss mice. Vero E6 cells (ATCC#1008) were maintained in Roswell Park Memorial Institute 1640 (RPMI-1640) medium supplemented with 10% fetal bovine serum (FBS), 2 mM l-glutamine, 10 mM HEPES, 100 UI/mL penicillin, and 100 µg/mL streptomycin (all reagents from Gibco, Grand Island, NY, USA) at 37°C and under a 5% CO_2_ atmosphere.

Additionally, blood trypomastigotes obtained from infected Swiss mice 10 days post-infection (dpi) were used for *in vitro* infection of Vero E6 cell monolayers. Trypomastigote forms derived from the cell-culture supernatant were used for parasite expansion in Vero E6 and infection of bone marrow-derived macrophages (BMDMs) and mice. Trypomastigote cultures were also used for EV purification and for *T. cruzi-antigen (TcAg)* preparation.

### *T. cruzi* EVs (EV Y)

Extracellular vesicles purified by tissue culture-derived trypomastigotes from *T. cruzi* strain Y were obtained as briefly described. Trypomastigotes collected from the supernatants of infected Vero E6 cells were washed three times in RPMI medium (2,600 × *g*, 10 min) and were incubated for 2 h at 37°C in RPMI (1 × 10^8^/mL) without FBS for spontaneous EV release. Trypomastigotes were removed by centrifugation (2,600 × *g* 10 min), and EV-containing supernatants were filtered through a 0.45-µm sterile membrane. EV purification was performed with total exosome isolation from cell-culture media reagent (Invitrogen, Carlsbad, CA, EUA) according to the manufacturer’s instructions. The EV Y pellet was suspended in phosphate-buffered saline (PBS).

The size, distribution, and concentration of EV Y were measured in a NanoSight LM10 instrument (Malvern Instruments Ltd, Malvern, UK). The data obtained were analyzed using Nanoparticle Tracking Analysis (NTA) software (version 3.1) with default settings, according to the manufacturer’s protocol. EV preparation was sampled twice to generate two independent dilutions and reduce the number of particles in the field of view below 200 detected tracks per image. From each sample, five videos (30 s each) were analyzed. All samples were measured using the same detection threshold (Figure S1 in Supplementary Material) (36).

### Experimental Design of *In Vivo* Experiments

C57BL/6 male mice were injected intraperitoneally with EV Y obtained from 10^6^ *T. cruzi* trypomastigotes. After 7 days, the animals were infected intraperitoneally with 5 × 10^3^ trypomastigote forms. Mice that were inoculated with EV Y or PBS but not infected and non-EV Y-treated mice infected with *T. cruzi* were used as controls. Parasitemia was monitored by counting the number of trypomastigotes in 5 µL of fresh blood collected from the tail vein as previously described (37). At day 12 post-infection (19 days after PBS or EV Y inoculation), the animals were anesthetized with ketamine (100 mg/kg) and xylazine (10 mg/kg) and sacrificed by cervical dislocation. The blood, heart, and spleen were collected. The blood was centrifuged at 1,300 × *g* for 10 min at room temperature, and the plasma was aliquoted and frozen for nitrite and cytokine measurement. The heart was fixed in 10% buffered formalin for histological analysis. Spleen cells were cultured with medium or total antigen of *T. cruzi* (TcAg) as previously described (38), and the supernatants were stored for the quantification of nitrite, cytokines, and prostaglandin E_2_ (PGE_2_) (Cayman, Ann Arbor, MI, USA). The 12th day after infection was chosen during this phase, NO production is elevated, the cells of the acquired immune response are already activated, and the host response begins to control parasitemia (39–41).

### Histological Analysis of the Heart

Heart halves were fixed in buffered formalin, dehydrated, and embedded in paraffin by a routine technique. Then, 5-µm-thick sections were stained with hematoxylin-eosin and analyzed by light microscopy. Cardiac parasitism was evaluated by counting the number of amastigote nests visualized at 1,000× magnification in six sections of the heart per animal, as previously described (42). The results are expressed as the mean of the number of amastigote nests per section. The data for each group are the mean ± SEM of five animals per group in two independents experiments.

### Peritoneal Lavage and Macrophages

To verify the *in vivo* effects of EV Y at the site of inoculation, we extracted peritoneal lavage and macrophages from mice in the four groups described below. Uninfected mouse samples were collected 7 days after PBS or EV Y (from 10^6^ *T. cruzi* trypomastigotes) inoculation. Infected groups were inoculated with PBS or EV Y, and after 7 days, they were infected with 5 × 10^3^ *T. cruzi*. The peritoneal lavage and macrophages were collected at 7 dpi. After anesthesia and cervical dislocation, 2 mL of cold PBS was injected into the mouse peritoneum, the peritoneum was massaged, and the fluid was collected. Peritoneal lavage fluid was centrifuged and frozen for nitrite quantification. Macrophage immunophenotyping was performed by flow cytometry.

### Peritoneal Macrophage Immunophenotyping

Cells were stained with a panel of immunophenotyping antibodies for 30 min at 4°C (anti-CD11b-FITC, anti-CD45-PE, anti-CD86-PECy5, anti-MHC-I-PE, anti-MHC-II-PECy5, and anti-F4/80-PE, all antibodies from BioLegend, Inc. CA, USA). Data were collected using an Accuri C5 flow cytometer (BD Biosciences, Franklin Lakes, NJ, USA) and analyzed by FlowJo software (Tree Star, Ashland, OR, USA). Compensation and isotype controls were also included. Gating strategies in SSC-A/SSC-H were used to exclude the doublets, and gating in forward and side scattering (FSC/SSC) allows the separation of macrophages from other small cells. Only CD11b^+^ cells (macrophages) were analyzed for the frequencies of other surface markers.

### Nitrite Quantification

The concentration of nitrite in spleen and macrophage cell-culture supernatants was directly determined by the Griess assay (43), which estimates total NO concentrations via nitrite analysis. Plasma and peritoneal lavage nitrite levels were evaluated by the cadmium Griess assay, as previously described (44).

### Cytokine Measurement by Flow Cytometry

Cytokine concentrations in plasma and spleen-cell culture supernatants were quantified by a Cytometric Bead Array (CBA)–Mouse Inflammation Kit, according to the manufacturer’s instructions (BD Biosciences, Franklin Lakes, NJ, USA). TNF-α, IL-6, IL-12p70, IFN-γ, MCP-1, and IL-10 flow cytometry measurements were performed on an Accuri C5 flow cytometer, and a total of 1,800 events were acquired for each sample. FCAP Array software was used for data analysis.

### Culture of Bone Marrow-Derived Macrophages

The differentiation of BMDMs from C57BL/6 mice was performed as previously described (45) using L929-cell-conditioned medium as a source of granulocyte/macrophage colony-stimulating factor (46). Fresh bone marrow cells were collected from two femurs and tibias in Dulbecco’s Modified Eagle’s Medium (DMEM) (Gibco, Grand Island, NY, USA) and depleted of erythrocytes with ammonium chloride. Cells were seeded in six-well tissue culture plates (Santa Cruz Biotechnology, Santa Cruz, CA, USA) at 2–3 × 10^5^ cells/mL and incubated at 37°C under a 5% CO_2_ atmosphere in DMEM supplemented with 25% FBS and 30% L929 cell-conditioned medium. Four days after the cells were seeded, 3 mL of fresh supplemented DMEM was added per well, and the cells were incubated for an additional 3 days. To harvest BMDMs, we discarded the supernatants and detached adherent cells. The cells were resuspended in supplemented DMEM that was used in all *in vitro* assays (10% FBS, 2 mM l-glutamine, 10 mM HEPES, 100 UI/mL penicillin, and 100 µg/mL streptomycin). The BMDM concentration was determined, and the cells were seeded in tissue culture plates for 4–12 h to ensure complete adherence.

### Experimental Design With Bone Marrow-Derived Macrophages

BMDM were seeded at a density of 2 × 10^5^ cells/well in 24-well plates with circular coverslips for a trypomastigote internalization assay and lipid body assay (47); in 48-well plates for a trypomastigote release assay; and in 96-wells plates for NO, PGE_2_, and cytokine quantification in the supernatants. After cells were adhered to the plates, they were incubated for 24 h at 37°C under a 5% CO_2_ atmosphere with EV Y obtained from 10^7^ *T. cruzi* trypomastigotes for parasite internalization, lipid body, and release assays. Next, the cultures were washed with PBS at 37°C to remove available EV Y, and trypomastigote forms from the *T. cruzi* Y strain were added to allow parasite–macrophage interaction as follows: 1 × 10^6^ parasites/well during 14 h of infection for the internalization and supernatant assays, 1 × 10^6^ parasites/well for 24 h for the lipid body assay, and 2 × 10^4^ parasites/well during 14 h of infection for the release assay. Non-internalized trypomastigotes were removed by three washes with PBS at 37°C. For the NO assay in supernatants, lipopolysaccharide (LPS from *Escherichia coli* 026:B6) was used as a positive control (Sigma, St. Louis, Missouri, EUA).

To investigate *T. cruzi* internalization, after 14 h of infection, we fixed macrophages with 100% methanol and stained them with Giemsa (Merck, Kenilworth, NJ, EUA). The coverslips were then removed from the plate and mounted on glass slides with Permount mounting medium (Fischer Chemical, Pittsburgh, PA, USA). Quantification of the number of amastigotes per macrophage was carried out using light microscopy where a total of 1,500–2,500 cells were randomly counted in each group. The internalization index was calculated by multiplying the percentage of infected cells by the mean number of parasites per infected cell (47, 48). The internalization of the control group, without EV Y, was considered 100%, and all internalization indices were normalized.

To determine the number of macrophage-released parasites, we collected the supernatants from 48-well plates daily and individually and subjected them to centrifugation (2,600 × *g*, 5 min, RT). Trypomastigotes were counted in a Neubauer chamber. The results are expressed as total number of *T. cruzi* by sample (41, 49).

### Osmium Tetroxide Staining for Lipid Bodies

To evaluate if EV Y induces the formation of lipid bodies in macrophages, we stained infected and uninfected cells with osmium tetroxide as previously described (50), with some modifications. Macrophages were fixed with formaldehyde solution (formalin) 3.7%. Cells were then washed with distilled water and stained with osmium tetroxide 1% in 0.1 M cacodylate buffer solution for 30 min. Then, the cells were washed three times with distilled water and incubated with thiocarbohydrazide 0.25% for 3 min, a step that promotes reduction of osmium and highly enhances lipid labeling. The thiocarbohydrazide was removed, and osmium tetroxide 1% in 0.1 M cacodylate buffer was reintroduced for 5 min. Finally, the cells were washed three times with distilled water and mounted on glass slides with Permount mounting medium. The cells were analyzed by light microscopy, and the number of lipid bodies per macrophage was counted in at least 1,000 macrophages in each group. In this staining process, osmium tetroxide binds to unsaturated lipids and is reduced to elemental osmium, an easily visible and permanent black marker.

### PGE_2_ Quantification

Prostaglandin E_2_ quantification in spleen and macrophage cell-culture supernatants was performed by a Prostaglandin E_2_ Express ELISA Kit from Cayman Chemical Co. (Ann Arbor, MI, USA). The test was performed according the instructions of the manufacturer, and the samples from infected mice or infected cells were diluted before analysis. The sensitivity of this assay was 15.6–2,000 pg/mL.

### Statistical Analysis

For column analysis, we used Student’s *t-*test to compare two groups and one-way analysis of variance (ANOVA) with Tukey’s post-test for more than three groups. To analyze grouped data, we used two-way ANOVA with Tukey’s or Sidak’s post-test. The values are presented as the mean ± SEM. The results were considered significant when *P* < 0.05. For statistical analysis, we used the program Prism (version 6.0, GraphPad Software Inc., San Diego, CA, USA).

## Results

### NTA of EVs Isolated From *T. cruzi* Strain Y

Extracellular vesicles spontaneously shed by tissue culture-derived trypomastigote forms from strain Y were analyzed by NTA and exhibited a profile compatible with that of previously described EVs of *T. cruzi* (15, 27, 51). In a representative analysis, EV Y had a mean diameter size of 136.33 nm with a SD of 86.3 nm and a mode of 94 (Figure S1 in Supplementary Material). Considering the range of EV Y sizes obtained, these suspensions are likely a mix of exosomes and plasma membrane-derived EVs. In this work, we will refer to those heterogeneous samples as EVs.

### *T. cruzi* EVs Increase Parasitemia and Cardiac Parasitism in C57BL/6 Mice

**Figure 1:**
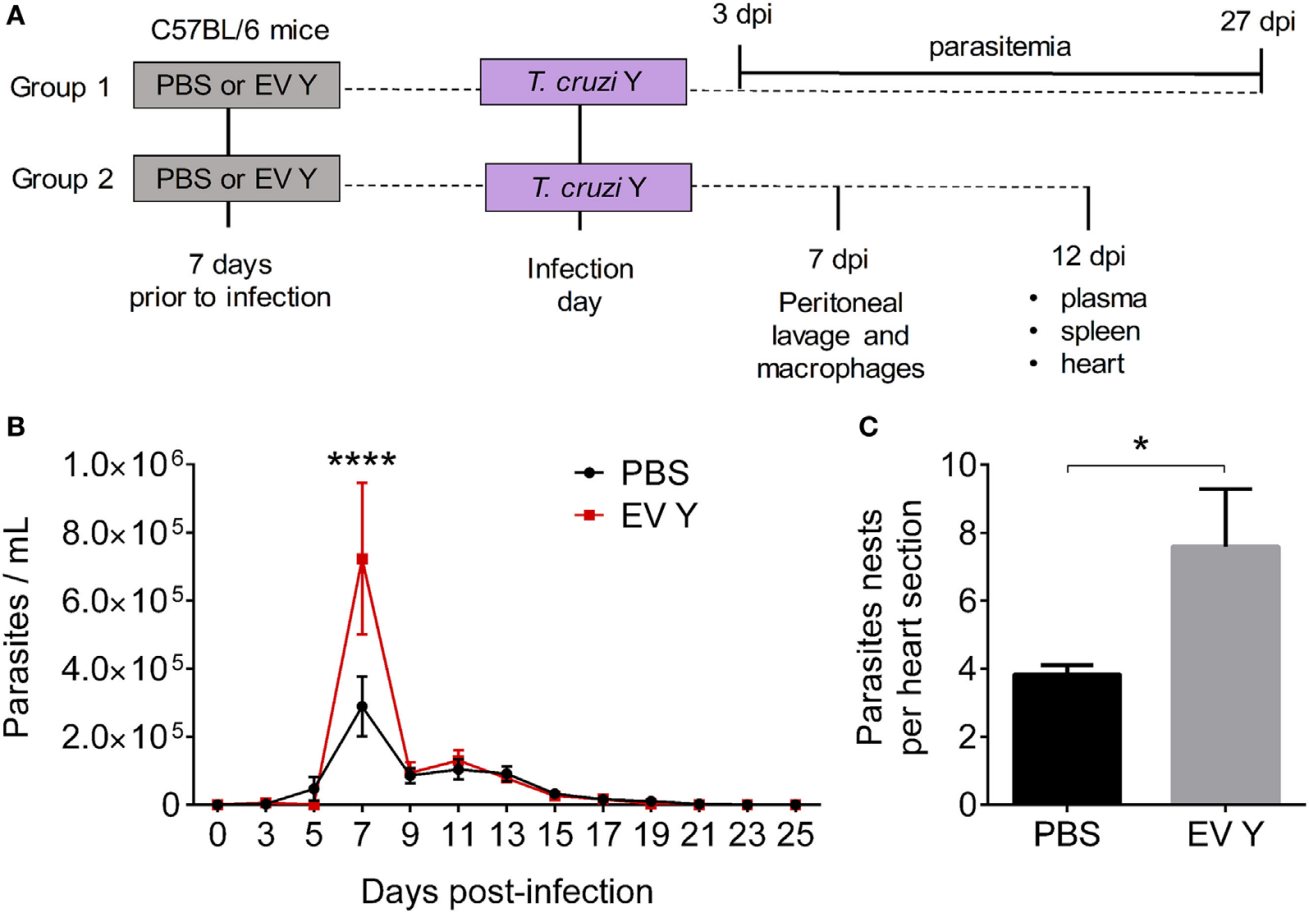
Previous inoculation of *Trypanosoma cruzi* extracellular vesicles (EVs) increases parasitemia and cardiac parasitism in C57BL/6 mice. C57BL/6 mice were inoculated with EVs derived from *T. cruzi* Y (EV Y) (i.p.). One week later, mice were infected with *T. cruzi* by the same route. **(A)** Experimental design for *in vivo* assays [days post-infection (dpi)]. **(B)** Parasitemia was quantified on alternating days as trypomastigotes per milliliter of blood. **(C)** Cardiac tissues were examined by hematoxylin and eosin staining on day 12 after *T. cruzi* infection. Parasitism was evaluated by counting the number of amastigote nests visualized at 1,000× magnification in six sections of the heart per animal. The results were expressed as the mean of the number of amastigote nests per section. The results are expressed as the mean ± standard error from of five mice per group in an experiment representative of two similar experiments. *P < 0.05; ****P < 0.0001 significantly different. Two-way ANOVA with **(B)** Sidak’s post-test and **(C)** Student’s *t-*test– GraphPad Prism.

Inoculation with PBS or EV Y prior to infection (Figure 1A) did not alter the parasitic load in blood in the beginning of the infection, specifically on 3 and 5 dpi. However, at 7 dpi, EV Y-inoculated mice showed 2.5 times more parasites in blood than did control PBS mice (Figure 1B, *P* < 0.0001). From 9 dpi, parasitemia constantly decreased, and by 17 dpi, the parasites in the blood appeared to be controlled in both PBS and EV Y-inoculated mice. Cardiac parasitism was also evaluated in PBS or EV Y-inoculated mice at 12 dpi. There were as many as two times more amastigote nests in the hearts of mice that had received EV Y prior to infection than in those of mice that had received only PBS prior to infection (Figure 1C, *P* < 0.05).

### Inoculation of *T. cruzi* EVs Prior to Infection Decreases the Levels of Plasma NO and TNF-α and Production of NO and Cytokines by Spleen Cells From Infected Mice

**Figure 2:**
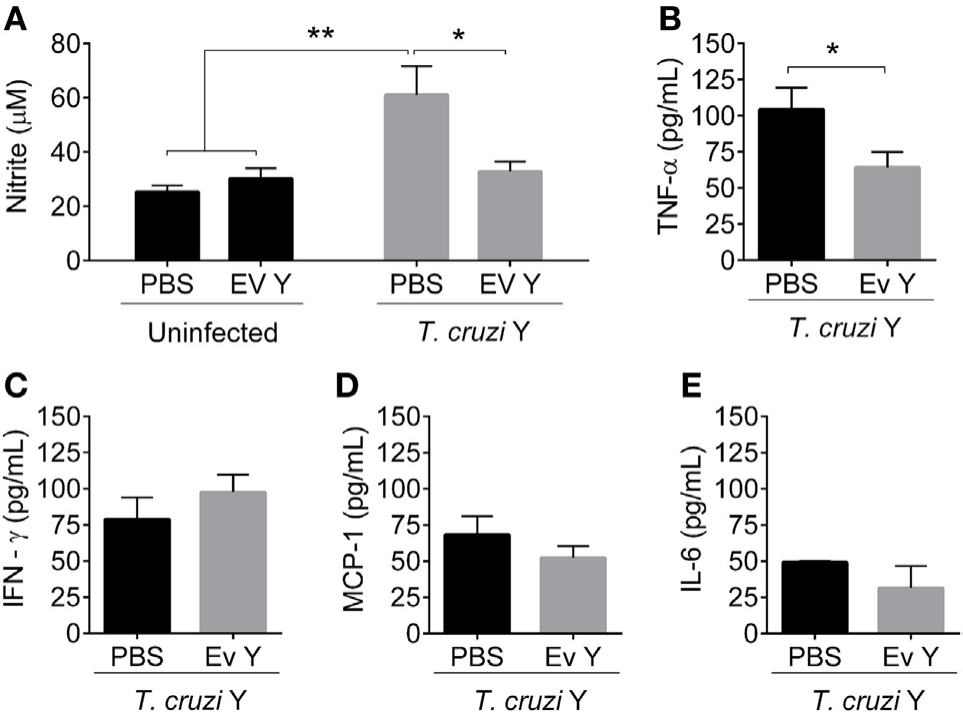
Inoculation of *Trypanosoma cruzi* extracellular vesicles (EVs) prior to infection decreases the plasmatic levels of nitric oxide and TNF-α in infected mice. C57BL/6 mice were inoculated with EVs derived from *T. cruzi* Y (EV Y) or phosphate-buffered saline (PBS, i.p.). One week later, the mice were infected with *T. cruzi* by the same route. Uninfected and PBS-inoculated mice were used as controls. Plasma was collected on day 12 after *T. cruzi* infection. **(A)** Nitrite in infected and uninfected mice was quantified by the cadmium Griess assay. Cytokines **(B)** TNF-α, **(C)** IFN-γ, **(D)** MCP-1, and **(E)** IL-6 were quantified by the Cytometric Bead Array. The results are expressed as the mean ± standard error from 5 to 9 mice per group in an experiment representative of two similar experiments. *P < 0.05; **P < 0.01 significantly different. Two-way ANOVA with **(A)** Sidak’s post-test and **(B–E)** Student’s *t-*test—GraphPad Prism.

Compared with control PBS inoculation in uninfected mice, EV Y inoculation in uninfected mice did not change NO in plasma (Figure 2A). As expected, compared with PBS in the absence of infection or EV Y inoculation, infection triggered a two-fold increase in circulating NO at 12 dpi (Figure 2A, *P* < 0.01). Although inoculation with EV Y had no effects on uninfected mice, inoculation with EV Y prior to infection downregulated the secretion of NO in mice infected at 12 dpi to half the NO levels in control mice (Figure 2A, *P* < 0.05).

Cytokine plasma levels (TNF-α, IFN-γ, MCP-1, IL-12p70, IL-10, and IL-6) in uninfected mice inoculated with PBS or EV Y were lower than the limit of detection of the assay. In infected mice, the levels of IL-12p70 and IL-10 were also below the detection threshold. However, despite the low levels of cytokines in the plasma of uninfected mice, *T. cruzi* infection resulted in the induction of high levels of cytokines TNF-α, IFN-γ, MCP-1, and IL-6 in plasma (Figures 2B–E). In addition, previous inoculation with EV Y in mice reduced the TNF-α level in plasma by ~50% compared with the level in PBS-inoculated infected mice (Figure 2B, *P* < 0.05). However, EV Y inoculation prior to infection did not alter the levels of IFN-γ, MCP-1, or IL-6 in the plasma of infected mice at 12 dpi compared with those in the plasma of PBS-inoculated infected mice (Figures 2C–E).

**Figure 3:**
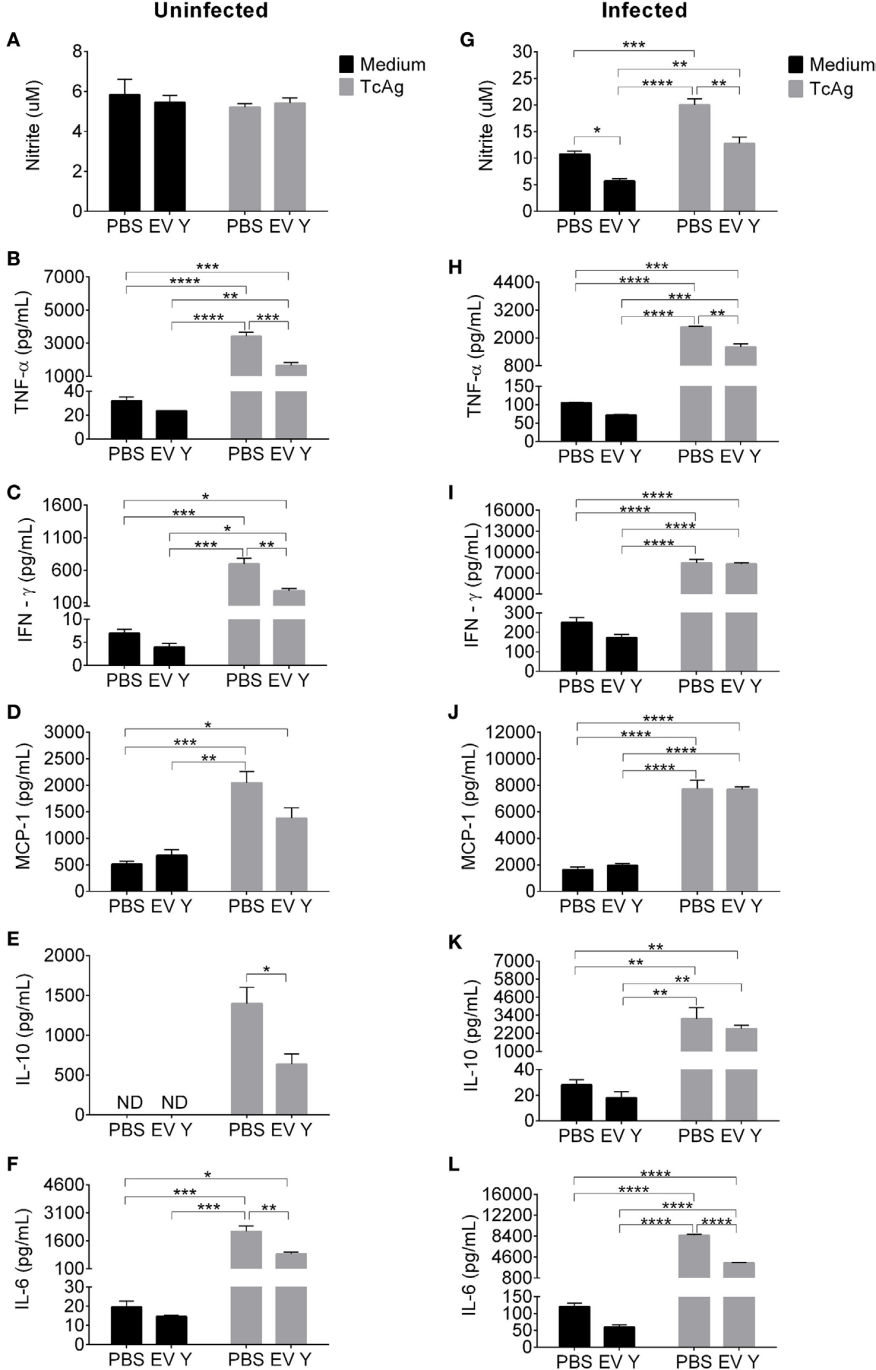
Inoculation of *Trypanosoma cruzi* extracellular vesicles (EVs) prior to infection decreases production of nitric oxide and cytokines by spleen cells from infected mice. C57BL/6 mice were inoculated with EVs derived from *T. cruzi* Y (EV Y) (i.p.). One week later, the mice were infected with *T. cruzi* (Y strain) by the same route. Infected, uninfected, and PBS-inoculated mice were used as controls. Spleens were harvested 12 days after *T. cruzi* infection (19 days after EV Y or PBS inoculation). The cells were cultured with medium or *T. cruzi* antigen (TcAg) for 48 h. **(A,G)** Nitrite in the supernatants of cultured spleen cells from uninfected mice (left column) and cultured spleen cells from infected mice (right column) were quantified by the Griess assay. Cytokines **(B,H)** TNF-α, **(C,I)** IFN-γ, **(D,J)** MCP-1, **(E,K)** IL-10, and **(F,L)** IL-6 in the supernatants of cultured spleen cells from uninfected and infected mice, respectively, were quantified by the Cytometric Bead Array. The results are expressed as the mean ± standard error from four mice per group in an experiment representative of two similar experiments. *P < 0.05; **P < 0.01; ***P < 0.001; **** *P* < 0.0001 significantly different (two-way ANOVA with Tukey’s post-test)—GraphPad Prism.

To assess the responsiveness of the immune cells from mice in the different experimental groups, we harvested spleen cells 12 days after *T. cruzi* inoculation (19 days after EV Y or PBS inoculation). Spleen cells from uninfected mice subjected to PBS or EV Y inoculation produced low and similar levels of NO when stimulated with TcAg (Figure 3A). Uninfected mouse spleen cells stimulated with TcAg resulted in increased levels of cytokines TNF-α, IFN-γ, MCP-1, IL-10, and IL-6 (Figures 3B–F). Spleen cells from uninfected mice inoculated with EV Y that were stimulated *in vitro* with TcAg showed at least two times lower levels of TNF-α, IFN-γ, IL-10, and IL-6 than did spleen cells from uninfected PBS-inoculated mice that were stimulated *in vitro* with TcAg (Figures 3B,C,E,F; *P* < 0.001, *P* < 0.01, *P* < 0.05, and *P* < 0.01, respectively).

As expected, compared with spleen cells from uninfected mice, spleen cells from *T. cruzi*-infected mice spontaneously produced more NO and cytokines when cultured with medium and even more when stimulated *in vitro* with TcAg (Figure 3). The spontaneous and TcAg-stimulated NO production by spleen cells isolated from infected mice inoculated with EV Y before infection was significantly lower than that by spleen cells from PBS-inoculated mice (Figure 3G, *P* < 0.05 and *P* < 0.01). In the same way, compared with spleen cells from PBS-inoculated mice that were stimulated *in vitro* with TcAg, spleen cells isolated from infected mice inoculated with EV Y before infection produced approximately half the levels of pro-inflammatory cytokines TNF-α and IL-6 when stimulated *in vitro* with TcAg (Figures 3H,L, *P* < 0.01 and *P* < 0.0001, respectively). However, EV Y inoculation *in vivo* was not always associated with a decrease in *ex vivo* production of cytokines. Even in the TcAg-stimulated spleen cells, inoculation with EV Y prior to infection did not alter the production of the pro-inflammatory cytokines IFN-γ (Figure 3I) and MCP-1 (Figures 3D,J). Notably, EV Y inoculation did not alter the production of the regulatory cytokine IL-10 (Figure 3K).

### Previous Inoculation of *T. cruzi* Extracellular Vesicles Decreases Production of Nitrite in the Peritoneal Lavage and Alters the Expression of Surface Activation Molecules in Macrophages Harvested From *T. cruzi*-Infected Mouse Peritoneum

**Figure 4:**
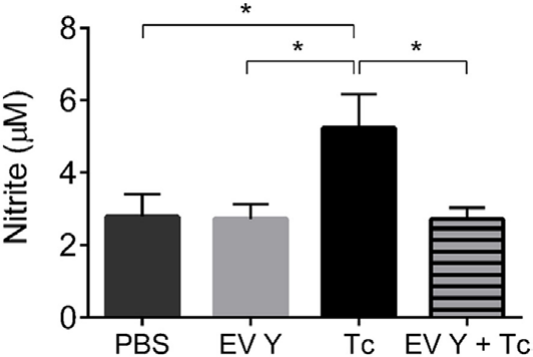
Previous inoculation of *Trypanosoma cruzi* extracellular vesicles (EVs) decreases production of nitric oxide in the peritoneal lavage of *T. cruzi*-infected mice. C57BL/6 mice were inoculated with EVs derived from *T. cruzi* Y (EV Y) (i.p.). One week later, the mice were infected with *T. cruzi* (Tc) by the same route or were inoculated with EV Y alone. Uninfected and PBS- or EV Y-inoculated mice were used as controls. On day 7 after *T. cruzi* infection and on day 7 after EV Y inoculation in uninfected animals, the peritoneum was washed, and nitrite was quantified in these fluids by the cadmium Griess assay. The results are expressed as the mean ± standard error from 4 to 9 mice per group in an experiment representative of two similar experiments. *P < 0.05 significantly different (one-way ANOVA with Tukey’s post-test)—GraphPad Prism.

The inoculation of PBS or EV Y *in vivo* and infection with *T. cruzi* were always performed by the intraperitoneal route. Hence, we decided to analyze the peritoneal environment through the quantification of NO in the peritoneal lavage and through phenotypical analyses of the macrophages present in the peritoneum of uninfected mice at 7 days after PBS or EV Y inoculation and in that of infected mice at 7 dpi (14 days after EV Y or PBS inoculation). In the uninfected groups, the NO levels were the same between PBS- or EV Y-inoculated mice (Figure 4). Compared with uninfected mice, PBS-inoculated mice showed a two-fold increase in NO production in the peritoneal lavage after *T. cruzi* infection. These increased levels of NO were not observed in mice that had received EV Y prior to infection (Figure 4).

**Figure 5:**
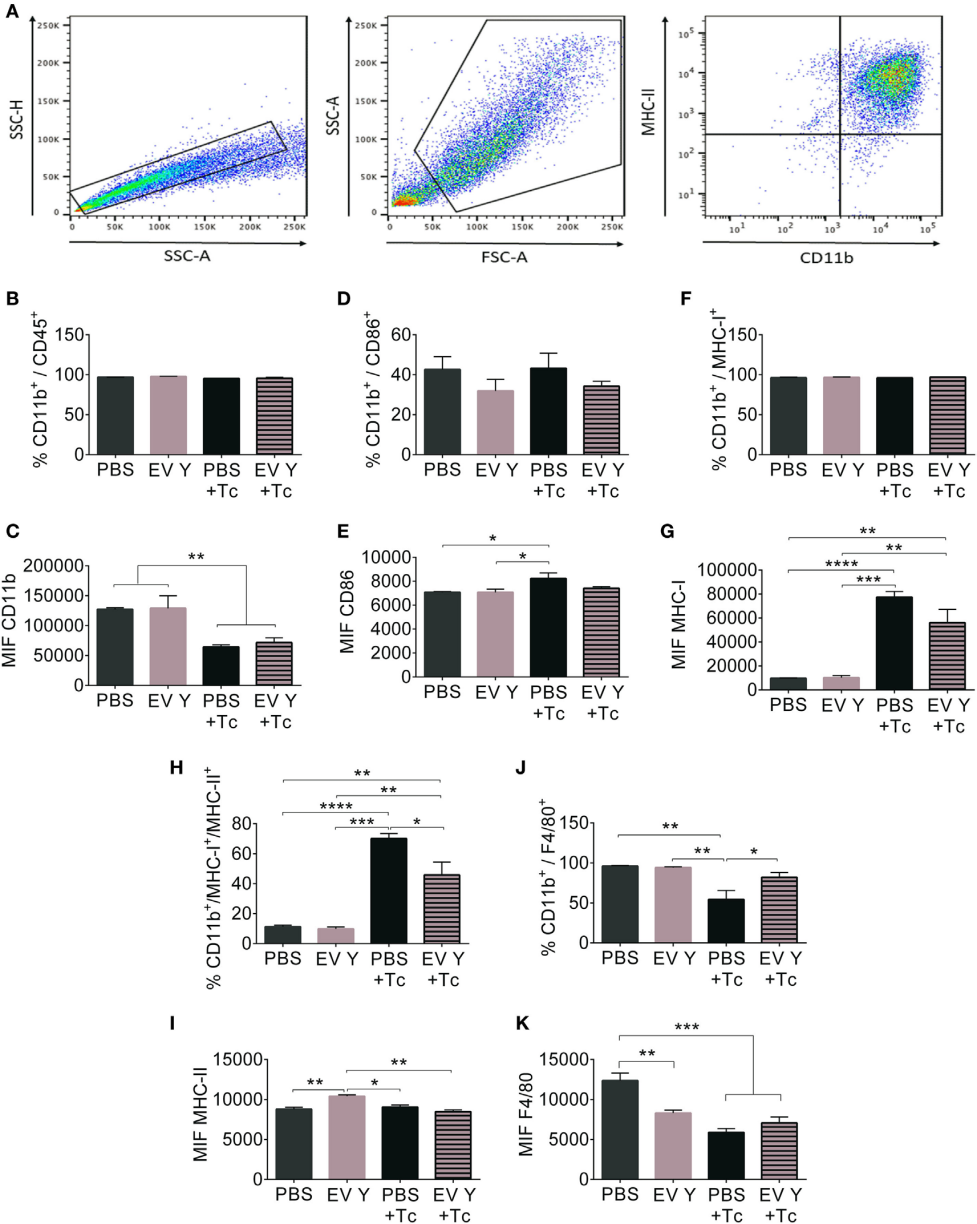
Previous inoculation of *Trypanosoma cruzi* extracellular vesicles (EVs) alters the expression and frequency of surface activation molecules in macrophages harvested from infected-mouse peritoneum. C57BL/6 mice were inoculated with extracellular vesicles derived from *T. cruzi* Y (EV Y) (i.p.). One week later, the mice were infected with *T. cruzi* by the same route or were inoculated with EV Y only. Infected, uninfected, and PBS-inoculated mice were used as controls. On day 7 after *T. cruzi* infection and on day 7 after EV Y inoculation in uninfected animals, peritoneum cells were harvested and analyzed by flow cytometry. **(A)** Gate strategy definition, **(B,C)** and the cells positive for the molecule CD11b were immunophenotyped for the frequency (%) and expression levels (median intensity of fluorescence (MIF)) of surface molecules **(D,E)** CD86, **(F,G)** MHC-I, **(H,I)** MHC-I/MHC-II, and **(J,K)** F4/80. The results are expressed as the mean ± standard error from 4 to 7 mice per group in an experiment representative of two similar experiments. *P < 0.05; **P < 0.01 significantly different (one-way ANOVA with Tukey’s post-test)—Graph Pad Prism.

The flow cytometry analysis of peritoneal macrophages is represented in Figure 5A. For this analysis, doublets were excluded, and peritoneal macrophages were identified according to size and complexity. From this gate, only CD11b^+^ cells were analyzed. Although most cells in the selected gate showed surface expression of the markers CD11b^+^ and CD45^+^, confirming the selection of the macrophage population (Figure 5B), the median fluorescence intensity (MIF) of CD11b after infection decreased by 50% in the PBS- and EV-inoculated groups (Figure 5C, *P* < 0.01). The percentage of cells expressing the co-stimulatory receptor CD86 was not significantly different between the groups (Figure 5D), but the cells from PBS-inoculated and infected mice showed more CD86 in macrophages than did cells from uninfected mice. Surprisingly, compared with the macrophages from uninfected groups, the macrophages from the group that had received EV Y before infection did not show increased CD86 (Figure 5E).

As expected, all cells exhibited the major histocompatibility complex class I (MHC-I) protein (Figure 5F), and infection increased MHC-I in macrophages by approximately sevenfold (Figure 5G, *P* < 0.0001).

The percentage of macrophages positive for MHC-II molecules was similar between the uninfected groups (PBS- and EV Y-inoculated). The infection triggered an increase in macrophages presenting MHC-II in mice inoculated with PBS before infection (*P* < 0.0001). Despite the increase in MHC-II in macrophages from EV Y-inoculated infected mice, this increase was 34% lower than that observed in macrophages from the PBS-inoculated and infected mice (Figure 5H, *P* < 0.05). When the levels of MHC-II were analyzed by MIF, macrophages from mice inoculated with EV Y and from mice that were uninfected showed higher MHC-II expression than did all the other mice, even infected mice (Figure 5I).

Concerning the analysis of F4/80 protein, the percentage of macrophages that tested positive for this protein was reduced by 50% in the PBS-inoculated and infected group. The infection did not induce a reduction in F4-80 expression in mice that had received EV Y before the infection (Figure 5J, *P* < 0.05). The uninfected group showed higher F4/80 expression levels than did the following three groups: EV Y-inoculated and uninfected, PBS-inoculated and infected, and EV Y-inoculated and infected groups (Figure 5K, *P* < 0.001).

### Macrophage Stimulation With *T. cruzi* EV Y Increases *T. cruzi* Infection and Trypomastigote Release From Infected Cells but Decreases Nitrite Production

**Figure 6:**
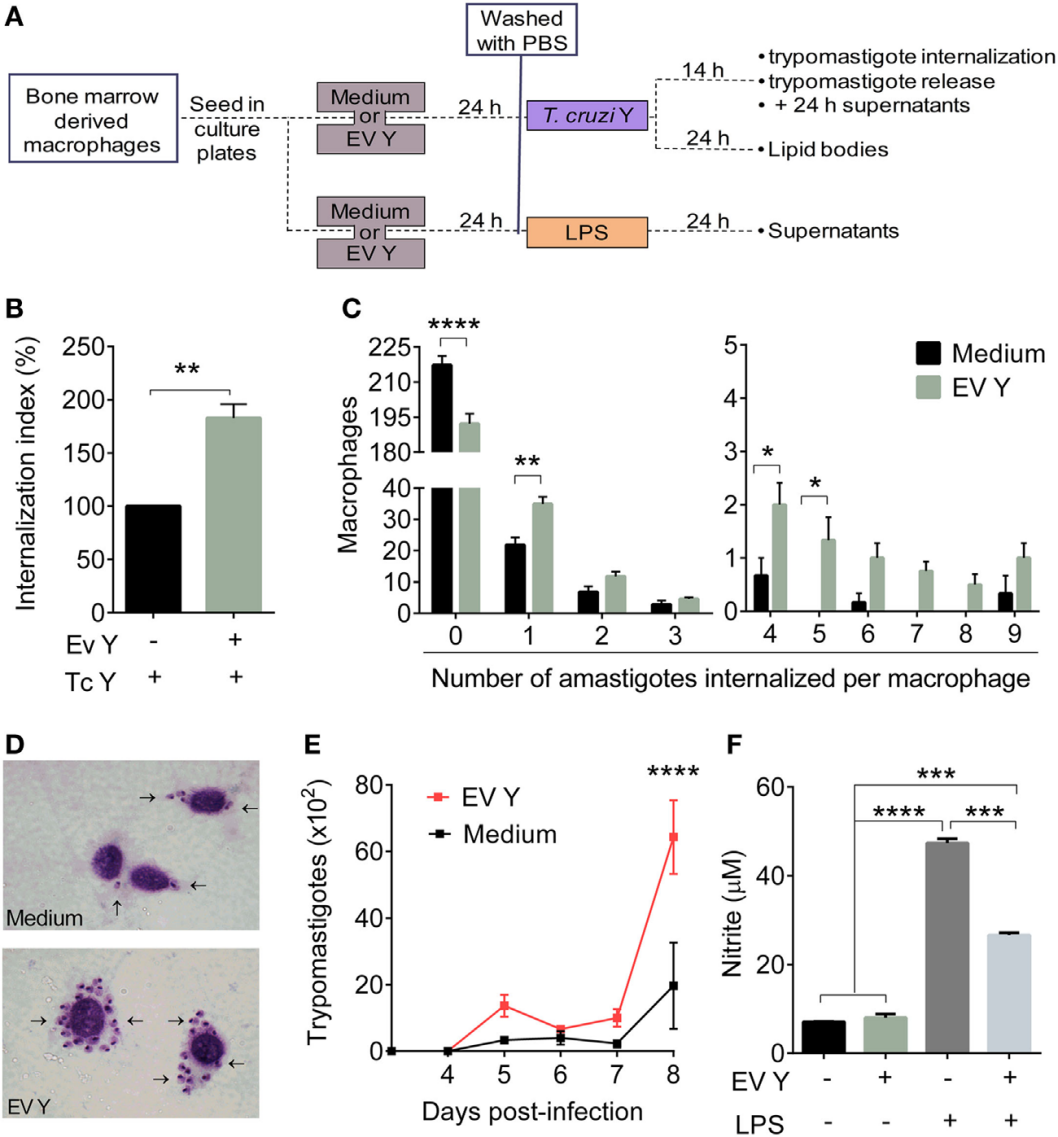
Macrophage *in vitro* stimulation with *Trypanosoma cruzi* extracellular vesicles (EVs) increases *T. cruzi* infection and trypomastigote release from infected cells but decreases nitric oxide release. Bone marrow-derived macrophages were incubated for 24 h with EVs derived from *T. cruzi* Y (EV Y) (i.p.). The cells were washed and infected with *T. cruzi* trypomastigote forms (0–1 parasites per macrophage for the trypomastigote release assay and 5 parasites per macrophage in the remaining assays) for 14 h or stimulated with 0.1 µg/mL of LPS for 24 h. After the clearance of non-internalized parasites, the cells were stained with Giemsa and analyzed by light microscopy. **(A)** Experimental design for macrophage *in vitro* assays **(B)** The internalization index was calculated considering the number of amastigotes internalized per macrophage. The internalization frequency of the control group, the absence of EV Y (black bar), was considered 100%. The gray bar represents macrophages stimulated with EV Y before *T. cruzi* infection. **(C)** The number of internalized amastigotes by macrophage in 250 counted cells. **(D)** Representative pictures of macrophages stained with Giemsa at 1000× magnification of infected cells in both groups (control and EV Y). Arrows indicate the internalized amastigotes in macrophages. **(E)** Daily number of trypomastigotes free in the supernatants. **(F)** Nitrites in the supernatants of macrophages cultured with LPS were quantified by the Griess assay. The results are expressed as the mean ± standard error from each group in triplicate analysis in an experiment representative of two similar experiments. *P < 0.05; **P < 0.01; ***P < 0.001; **** *P* < 0.0001 significantly different. **(A)** Student’s *t-*test, **(B,D)** two-way ANOVA with Sidak’s post-test and **(E)** one-way ANOVA with Tukey’s post-test—GraphPad Prism.

In the *in vitro* assays, BMDMs were incubated with EV Y for 24 h, and after removal of EV Y, the cells were infected with trypomastigotes (Figure 6A). This previous contact of the macrophages with EV Y promoted a significant increase (82%) in the number of internalized amastigotes, as assessed by the internalization index (Figure 6B, *P* < 0.01). The mean number of amastigotes in 250 macrophages showed that macrophages previously stimulated with EV Y were more infected and presented a higher number of internalized amastigotes (Figures 6C,D).

To verify the infection efficiency rate, we measured the release of trypomastigotes daily in the supernatant of infected macrophage cultures. The macrophages that had contact with EV Y before infection released three times more trypomastigotes than did the macrophages cultured only with medium, especially on the eighth day after the infection (Figure 6E, *P* < 0.0001).

To evaluate the modulation of NO production by EV Y *in vitro*, as BMDM were not activated by *in vivo* events such as chemotaxis gradients or cytokines, we stimulated BMDMs with LPS. LPS is a classical activator of macrophages by TLR4 stimulation; in response to LPS, macrophages produce NO, among other things (positive control) (45). Macrophages were incubated with EV Y for 24 h, and after removal of EV Y, cells were stimulated with LPS for another 24 h. Incubation with EV Y alone did not induce NO production by macrophages. By contrast, LPS induced high levels of NO release by macrophages (*P* < 0.0001). However, compared with macrophages stimulated with LPS alone, macrophages incubated with EV Y and then stimulated with LPS produced half of the level of NO (Figure 6F, *P* < 0.001).

### EV Y Stimulation Induces Macrophage Lipid Body Formation and PGE_2_ Production and Reduces Pro-Inflammatory Cytokine Production

**Figure 7:**
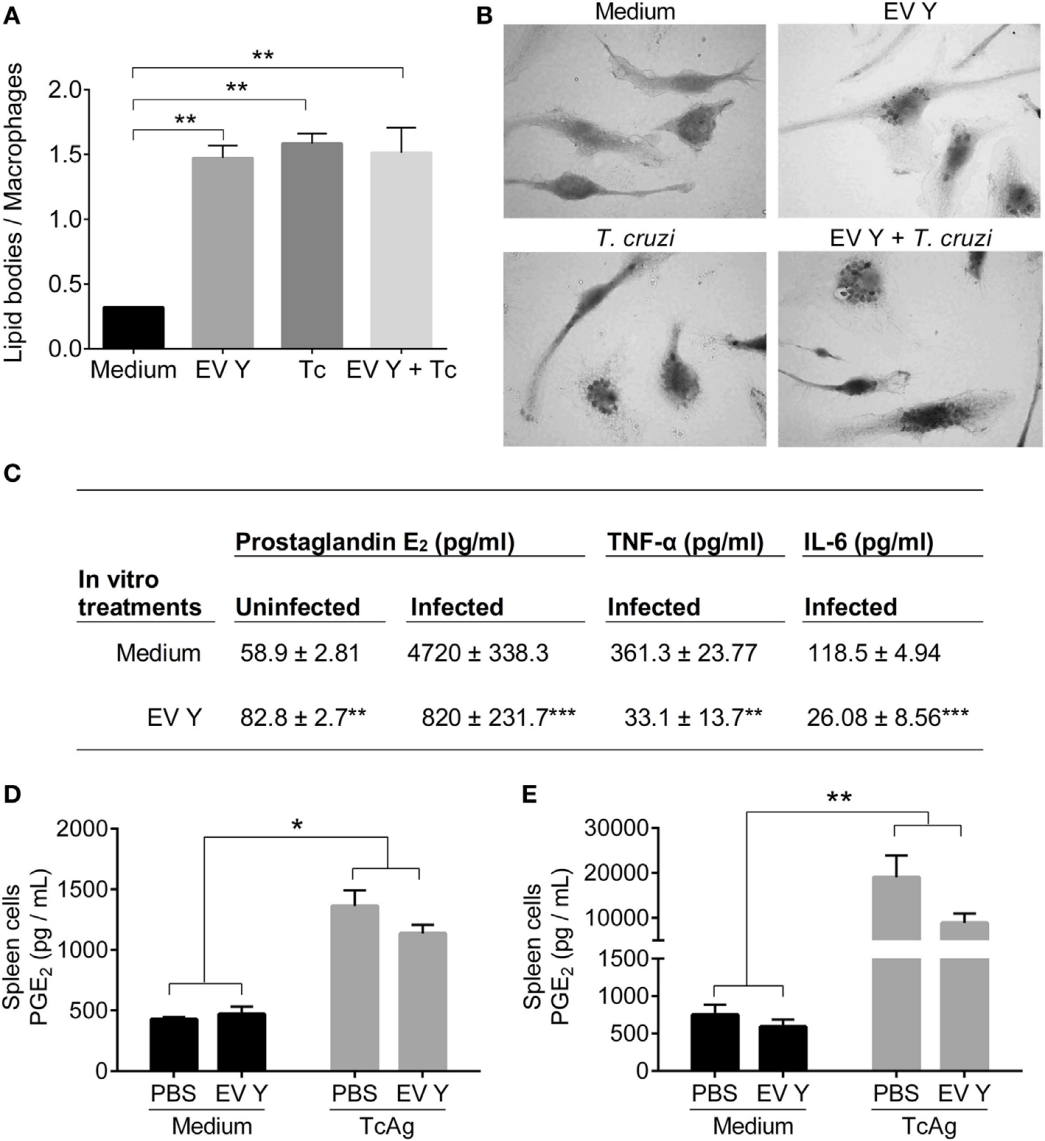
*Trypanosoma cruzi* extracellular vesicles (EVs) induce lipid body formation and prostaglandin E_2_ (PGE_2_) production by macrophages (*in vitro*) but not by spleen cells (*ex vivo*). Bone marrow-derived macrophages were incubated for 24 h with EVs derived from *T. cruzi* Y (EV Y) (i.p.). The cells were washed and infected with trypomastigote forms of *T. cruzi* (5 parasites per macrophage). After 24 h, non-internalized parasites were removed. **(A)** The cells were stained with osmium tetroxide and analyzed by light microscopy. The results are expressed as the mean ± standard error from each group in 250 cells counted in triplicate analysis in an experiment representative of two similar experiments. **(B)** Representative pictures of macrophage staining with osmium tetroxide at 1,000× magnification of uninfected cells (without and with EV Y-upper panel) and infected cells in both groups (control and EV Y- bottom panel). The lipid bodies are visible as black markers inside the macrophages. **(C)** PGE_2_ was quantified by ELISA, and cytokines (TNF-α and IL-6) were quantified by CBA in the supernatant culture of macrophages (uninfected and infected cells) after 24 h of infection. The results are expressed from two experiments. C57BL/6 mice were inoculated with EV Y (i.p.) and infected with *T. cruzi* by the same route 1 week later. Infected, uninfected, and PBS-inoculated mice were used as controls. Spleens were harvested 12 days after *T. cruzi* infection. The cells were cultured with medium or *T. cruzi* antigen (TcAg) for 8 h. **(D,E)** PGE_2_ was quantified by ELISA in the supernatants of cultured spleen cells from uninfected and infected mice. The results are expressed as the mean ± standard error from four mice per group in one experiment. *P < 0.05; **P < 0.01 significantly different (one-way ANOVA with Tukey’s post-test)—GraphPad Prism.

To verify the influence of EV Y in lipid body formation in macrophages, we incubated BMDMs with EV Y for 24 h, and after removal of EV Y, the cells were infected with trypomastigotes for another 24 h. Non-stimulated or EV Y-stimulated cells were analyzed. Osmium tetroxide staining was used to detect lipid bodies, which resemble black marks in the cytoplasm (Figure 7B). The infection increased the formation of lipid bodies in macrophages by fourfold, and previous incubation with EV Y did not alter these lipid body levels. Interestingly, incubation of macrophages with EV Y alone was sufficient to induce lipid bodies in macrophages (Figure 7A, *P* < 0.01).

Additionally, we assessed PGE_2_ production by macrophages exposed to EV Y. In addition to lipid body formation, EV Y-stimulated macrophages showed higher PGE_2_ production than did non-stimulated macrophages (Figure 7C, *P* < 0.01). Previous contact with EV Y markedly reduced PGE_2_ production in uninfected cells compared with that in infected cells cultured with medium (*P* < 0.001). EV Y also decreased TNF-α (*P* < 0.01) and IL-6 (*P* < 0.001) production in infected macrophages (Figure 7C).

To assess the *in vivo* effect of EV Y on PGE_2_ production, we harvested spleen cells from mice in the different experimental groups 12 days after *T. cruzi* inoculation (19 days after EV Y or PBS inoculation) and cultured them *in vitro* with medium or stimulated them with TcAg for 8 h. Spleen cells from uninfected and infected mice stimulated with TcAg produced more PGE_2_ than did non-stimulated cells (Figures 7D,E, *P* < 0.05). In contrast to macrophages exposed to EV Y *in vitro*, macrophages and spleen cells inoculated with EV Y *in vivo* did not exhibit altered PGE_2_ production in these animals at this time point in either uninfected or infected mice (Figures 7D,E).

## Discussion

The pathogenesis of Chagas disease is complex and involves many interactive pathways between hosts and parasites (52). In this context, only in the last decade has the role of EVs released by *T. cruzi* been recognized; however, the mechanisms by which EV Y exert their pathological effects are incompletely understood (53). Elucidating the underlying mechanism of the pathogenic effects of EVs shed by *T. cruzi* is pertinent to better understand the parasite–host relationship.

In 2009, a study reported that inoculation with EVs shed by *T. cruzi* prior to infection in susceptible BALB/c mice increased cardiac parasitism and accelerated mortality rates, without a significant increase in blood parasitemia (26). Herein, we explored the role of EV Y in experimental acute murine infection using resistant C57BL/6 mice and the Y strain of *T. cruzi*. Animal inoculation with EV Y before infection with trypomastigote forms led to a transient but substantial increase in the blood parasitic load at the beginning of the infection (7 dpi). A few days later, at 12 dpi, when the blood parasitic load was similar between the control and the EV Y-inoculated groups, amastigote parasitism in cardiac tissue was higher in mice that had received EV Y prior to infection. However, inoculation with EV Y before infection did not lead to mortality among the animals.

The role of EV Y in intercellular communication between host cells and *T. cruzi* has been considered in recent years (54). Bayer-Santos et al. showed that EVs released by *T. cruzi* are endocytosed by cells and reach the cytoplasm where they can eventually release their content (15). In another study, Neves et al. demonstrated that EVs shed by *T. cruzi* Y strain act in macrophages by increasing the adherence of the parasite, while EVs released by *T. cruzi* CL-Brener strain enhance macrophage infection by this strain through acid phosphatase activities (55). Garcia-Silva et al. showed that EV cargo promotes the life-cycle transition of epimastigotes into trypomastigotes and can be transferred between parasites and susceptible mammalian cells, increasing their infection susceptibility (28). The effects of EVs shed by parasites in infections are not exclusive to *T. cruzi*. In fact, in murine infections with *Leishmania major* and *Leishmania donovani*, preinoculation with EVs from these parasites resulted in increased edema and parasitism in the hind legs of mice (23). *In vitro*, the communication between parasites and macrophages through parasite EVs was also described in *Leishmania* by Silverman et al., who showed that parasite EVs induced production of IL-8 but not TNF-α by macrophages, which led to immunosuppression in the host and prepared cells for *Leishmania* infection (56).

During the acute phase of infection, the recognition of *T. cruzi* by Toll-like receptors on macrophages (57) triggers production of TNF-α and IL-12, which in turn activates other cells, such as natural killers and lymphocytes, to produce IFN-γ (58). Hence, IFN-γ activates macrophages and controls intracellular parasite growth through the production of NO by the enzyme inducible nitric oxide synthase (iNOS) and reactive oxygen molecules (6, 59, 60). NO has a trypanocidal effect (39) as it reacts with superoxide (O_2_^−^) to produce the very toxic molecule peroxynitrite (ONOO^−^), which leads to oxidation of thiol groups and nitrosylation of parasitic amino acids and proteins (61, 62). NO also modulates the effector immune response by regulating/enhancing inflammation during *T. cruzi* infection (60).

Although infection with *T. cruzi* triggered NO production assessed in plasma, mice that were inoculated with EV Y prior to *T. cruzi* challenge did not show a corresponding increase in the plasmatic levels of the trypanocidal molecule NO. In the peritoneum, the location of EV Y and *T. cruzi* inoculation, the levels of NO in mice that had received EV Y prior to infection were also lower than those in control mice and were similar to the levels in uninfected mice. The same phenomenon occurred *in vitro* when spleen cells from mice that had received EV Y *in vivo* before infection were stimulated with TcAg. Taken together, these data show that intraperitoneal inoculation with EV Y was able to alter the responsiveness of immune cells both locally and systemically. Inoculation of EV Y without subsequent infection did not stimulate NO production in plasma, peritoneal lavage or spleen-cell supernatants. These results suggest that EV Y stimulation had no direct trypanocidal activity in host macrophages, but it modulates these cells, creating a favorable environment for the parasite with lower NO production, which probably contributes to the higher parasitemia observed in mice treated with EV Y before infection.

The lower production of NO in infected mice induced by previous contact with EV Y partially corroborates the results of Torrecilhas et al. (26). They described the low activity of iNOS in the heart tissue of infected BALB/c mice primed with *T. cruzi* vesicles, but there was no significant difference in NO levels in the supernatants of spleen-cell cultures between vesicle-treated and control animals. In a chronic study developed by Nogueira et al., spleen cells from infected mice at 180 dpi were cultured *in vitro* with vesicles from different *T. cruzi* strains and responded with increased production of NO (51). However, a major difference between these studies should be mentioned and has to be taken into consideration; in the acute infection model shown here and by Torrecilhas et al., EV Y was administered *in vivo* before infection (26), whereas in the chronic model, the vesicles were added *in vitro* to the spleen cells of chronically infected animals (51).

Although inoculation was always performed in the peritoneal cavity, our study provided further evidence for the systemic effect of EV Y in infected mice; modulation of the innate immune response was demonstrated by the low plasmatic levels of TNF-α and by low production of TNF-α and IL-6 by spleen cells stimulated *in vitro* with TcAg. During the first weeks of infection, the pro-inflammatory cytokines TNF-α and IL-6 are required for activation of inflammatory cells and for a subsequent efficient Th1 immune response against the parasite (7, 40, 63). Low production of these cytokines induced by previous contact with EV Y probably favored the infection. Additionally, inoculation with EV Y did not change production of the regulatory cytokine IL-10 by spleen cells from infected mice stimulated *in vitro* with TcAg. The high concentration of IL-10 during the acute phase is associated with failed control of infection (64), and as shown here, EV Y altered the balance between the pro-inflammatory/regulatory cytokines toward an immunoregulatory profile. Consistent with these results, Torrecilhas et al. showed increased expression of IL-10 and IL-4 mRNA in heart tissue from BALB/c mice treated with *T. cruzi* vesicles before infection and showed low production of TNF-α by macrophages stimulated *in vitro* with *T. cruzi* vesicles (26).

EV Y inoculation without infection did not stimulate the production of NO, but spleen cells from uninfected mice inoculated with EV Y and stimulated *in vitro* with TcAg produced lower levels of TNF-α, IFN-γ, IL-10, and IL-6 than did spleens cells from control mice. Although these cells never had contact with the parasite, they responded to the TcAg stimulus by producing cytokines, albeit at a lower level than the infected animals. Even in these non-primed cells, EV Y exercised immunomodulatory effects by decreasing the production of cytokines after stimulus.

The phenotypic analysis of peritoneal macrophages showed a high percentage of cells positive for CD11b and CD45, confirming that the peritoneal population analyzed was composed of macrophages. CD11b belongs to the integrin β2 family and plays an important role in the migration of macrophages (65), while CD45, a protein tyrosine phosphatase expressed on all cells of hematopoietic origin, is associated with the adhesion process (66). The reduction in the amount of these superficial molecules observed in both the EV Y and control groups may be related to the internalization mechanisms of these molecules in activated macrophages (67).

Macrophages present antigens to lymphocytes through peptides associated with MHC-I (endogenous antigens) and MHC-II (exogeneous antigens) molecules (68). Additionally, co-stimulatory molecules, such as CD86 (or B-7), are necessary for efficient activation of lymphocytes by macrophages (69). EV Y inoculation before infection did not alter the percentage and expression of MHC-I in peritoneal macrophages. However, compared with PBS inoculation before infection, EV Y inoculation before infection resulted in a lower percentage of macrophages that were positive for MHC-II. Extrapolating these data, the results indicate that previous contact with EV Y might interfere with the presentation of phagocytized antigens by macrophages to lymphocytes. Reinforcing this interpretation, peritoneal macrophages from mice that received EV Y before infection showed less CD86 than did macrophages from mice that received PBS before infection. Interestingly, although EV Y inoculation without infection did not change the percentage of MHC-II positive cells, the expression or the amount of MHC-II in these cells was slightly higher than that in all the other cells, as assessed by the MIF.

The F4/80 molecule, frequently used as a marker for mouse macrophages, has been characterized as a member of the epidermal growth factor (EGF)-transmembrane 7 (TM7) family, and there is evidence for its role in the induction of immunological tolerance (70). The induction of tolerance instead of an inflammatory environment could be beneficial to the parasite. The percentage of macrophages positive for F4/80 decreased in PBS-infected mice but not in mice that had received EV Y before infection. This downregulation of F4/80 in macrophages indicates how EV Y could be modulating the immune response toward a less inflammatory environment. Surprisingly, the amount of F4/80 was lower in macrophages inoculated with EV Y without infection than in macrophages inoculated with PBS without infection.

Considering the *in vivo* immunomodulatory effects exerted by EV Y described here, we investigated the specific role of EV Y using *in vitro* assays with BMDMs. Macrophages that had contact with EV Y before infection exhibited enhanced internalization of *T. cruzi* trypomastigotes. In the initial steps of infection, in addition to the adherence and invasion process, *T. cruzi* needs to evade the trypanocidal response of macrophages to replicate and continue its life cycle (13). In addition to a higher internalization rate, EV Y enhanced the release of parasites by macrophages. Moreover, corroborating our *in vivo* findings, the effect of EV Y in decreasing macrophage responsivity was confirmed by the reduction in NO production in macrophage cultures activated with LPS, a classic TLR4 agonist and inducer of NO production (45).

In 2003, Melo et al. demonstrated for the first time that *T. cruzi* infection triggers the formation of lipid bodies in macrophages (71), and this event is directly related to PGE_2_ synthesis by macrophages (71, 72). They also showed that the enzyme cyclooxygenase-2 (COX-2) is co-localized with lipid bodies in infected macrophages, further strengthening these structures as substrates that are necessary for PGE_2_ synthesis (72). As expected, in our assays, infection with *T. cruzi* induced lipid body formation in macrophages. However, the contact of macrophages with EV Y without infection also induced lipid bodies in those cells but did not cause additional lipid body formation in infected macrophages. In fact, D’Avila et al. demonstrated that uninfected macrophages from the infected group also showed higher lipid body formation than did macrophages from the control group; the authors described this observation as bystander amplification (72). These results provide new insights to explain this phenomenon where EVs shed by *T. cruzi* induce lipid body formation in uninfected macrophages.

Additionally, EV Y alone induced production of PGE_2_ by macrophages. PGE_2_ production by activated macrophages occurs at the beginning of *T. cruzi* infection by the COX enzyme and plays a crucial role during the development of the infection (73–75). In macrophages, high concentrations of PGE_2_ diminished the production of pro-inflammatory cytokines and reduced antigen presentation and production of free radicals in these cells (76, 77). In this way, PGE_2_ is associated with the immunosuppression observed in infected mice with reduced lymphocyte proliferation and TNF-α production (73, 78). Moreover, uptake of apoptotic cells by macrophages during *T. cruzi* infection potentiates release of PGE_2_ and TGF-β by these cells, consequently decreasing production of NO, allowing intracellular parasite survival and growth (79). Furthermore, pharmacological inhibition of COX-2 using aspirin in peritoneal macrophages of BALB/c mice diminished *T. cruzi* internalization with consequent increases in IL-1β, NO and lipoxin production (80). Hence, increased production of PGE_2_ in macrophages that had contact with EV Y supports reduced production of pro-inflammatory cytokines (TNF-α) and NO that occurred in plasma and spleen-cell supernatants (TNF-α and IL-6) from mice inoculated with EV Y before infection. However, the release of PGE_2_ by infected macrophages after 24 h was significantly lower in cells that had contact with EV Y prior to infection than in cells without contact prior to infection. Although the EV Y induced lower levels of PGE_2_ produced by infected macrophages, the pro-inflammatory status of these cells was already downregulated, as shown by the abrupt reduction in release of the cytokines TNF-α and IL-6. Therefore, the immune modulation exerted by PGE_2_ that was induced by EV Y seems to be important specifically in the beginning of infection. In support of this hypothesis, *in vitro* production of PGE_2_ by spleen cells harvested from mice at 12 dpi did not change between animals inoculated with PBS or EV Y prior to infection, while the pro-inflammatory cytokines were downregulated in the same cells from animals inoculated with EV Y. Moreover, Moraes et al. showed that during the first 48 h of *T. cruzi* infection in H9c2 cells, COX-2 expression and activity are modulated by the parasite, leading to the control of the pro-inflammatory environment in infected cells (81). Considering that EVs shed by *T. cruzi* alter the genic expression of host cells (28), we hypothesize that EV Y could be modulating the expression and activity of COX-2.

In conclusion, our results indicate that EV Y exerts effects on host cells before infection, making the environment more favorable to *T. cruzi* in the first moments of infection. We showed that EVs released by *T. cruzi* modulate the immune response *in vivo* and demonstrated that this modulation occurs *in vitro* by direct effects on macrophages. These effects induce formation of lipid bodies and production of PGE_2_ and create a more favorable environment for parasite infection, with a reduction in inflammatory cytokines and the trypanocidal molecule NO. These results support the role of *T. cruzi* EVs in the complex pathogenesis of the acute phase of Chagas disease and provide new insights for a better understanding of the parasite–host relationship.

## Ethics Statement

This study was carried out in accordance with the recommendations of the Guide for the Care and Use of Laboratory Animals of the Brazilian National Council of Animal Experimentation. The protocol was approved by the Committee on the Ethics of Animal Experiments at Londrina State University (CEEA, process number 11/2015- 7045.2015.06). The use of Swiss mice for the maintenance of the parasite strain was also approved for the Committee on the Ethics of Animal Experiments at Londrina State University (CEEA, process number 28.841.2016.41).

## Author Contributions

MM, SG, PW, and PF contributed to conception and design of the study. PM standardized and performed extracellular microvesicles isolation. MM, AM, NZ, BL and VT performed animal and cell culture experiments. AO contributed to establishment of lipid body staining and analysis. MM, PW, and PF wrote the first draft of the manuscript. All authors contributed to manuscript revision. All authors read and approved the submitted version.

## Conflict of Interest Statement

The authors declare that the research was conducted in the absence of any commercial or financial relationships that could be construed as a potential conflict of interest. The reviewer HC declared a shared affiliation, though no other collaboration, with several of the authors, ML, PM, SG, PW, to the handling Editor.
