# Supplementary material for: Extracellular Vesicles Shed By Trypanosoma cruzi Potentiate Infection and Elicit Lipid Body Formation and PGE2 Production in Murine Macrophages
Source: Front Immunol. 2018 Apr 27;9:896. doi: 10.3389/fimmu.2018.00896 (PMC5934475; doi:10.3389/fimmu.2018.00896)
Supplement: Supplementary file 1 [file Image_1.PDF]

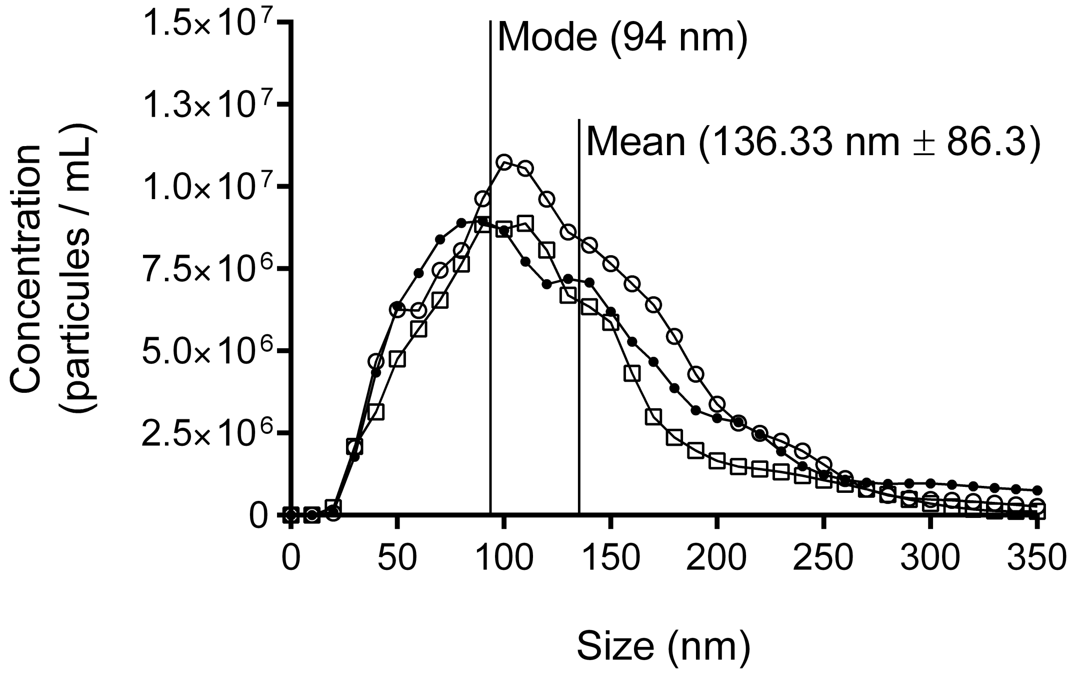

Supplementary Figure 1. Nanoparticle tracking analysis of the extracellular vesicles isolated from *T. cruzi* Y. Concentration (particles/mL) and size distribution (nm) for extracellular vesicles spontaneously shed by tissue culture-derived trypomastigote forms of *T. cruzi* Y (EV Y) measured in a NanoSight LM10 instrument (Malvern Instruments Ltd). The data are representative of 3 independent experiments.
